# Genomic Balancing Act: deciphering DNA rearrangements in the complex chromosomal aberration involving 5p15.2, 2q31.1, and 18q21.32

**DOI:** 10.1038/s41431-024-01680-1

**Published:** 2024-09-10

**Authors:** Zain Dardas, Dana Marafi, Ruizhi Duan, Jawid M. Fatih, Omnia F. El-Rashidy, Christopher M. Grochowski, Claudia M. B. Carvalho, Shalini N. Jhangiani, Weimin Bi, Haowei Du, Richard A. Gibbs, Jennifer E. Posey, Daniel G. Calame, Maha S. Zaki, James R. Lupski

**Affiliations:** 1 Department of Molecular and Human Genetics, Baylor College of Medicine, Houston, TX USA; 2 Department of Pediatrics, Faculty of Medicine, Kuwait University, Safat, Kuwait; 3 Department of Pediatrics, Faculty of Medicine Ain Shams University, Cairo, Egypt; 4 Pacific Northwest Research Institute, Seattle, WA USA; 5 Human Genome Sequencing Center, Baylor College of Medicine, Houston, TX USA; 6 Baylor Genetics, Houston, TX USA; 7 Texas Children’s Hospital, Houston, TX USA; 8 Section of Pediatric Neurology and Developmental Neuroscience, Department of Pediatrics, Baylor College of Medicine, Houston, TX USA; 9 Department of Clinical Genetics, Human Genetics and Genome Research Institute, National Research Centre, Cairo, Egypt; 10 Department of Pediatrics, Baylor College of Medicine, Houston, TX USA

**Keywords:** Cytogenetics, Neurodevelopmental disorders, Genetics research

## Abstract

Despite extensive research into the genetic underpinnings of neurodevelopmental disorders (NDD), many clinical cases remain unresolved. We studied a female proband with a NDD, mildly dysmorphic facial features, and brain stem hypoplasia on neuroimaging. Comprehensive genomic analyses revealed a terminal 5p loss and a terminal 18q gain in the proband while a diploid copy number for chromosomes 5 and 18 in both parents. Genomic investigations in the proband identified an unbalanced translocation t(5;18) with additional genetic material from chromosome 2 (2q31.3) inserted at the breakpoint, pointing to a complex chromosomal rearrangement (CCR) involving 5p15.2, 2q31.3, and 18q21.32. Breakpoint junction analyses enabled by long-read genome sequencing unveiled the presence of four distinct junctions in the father, who is a carrier of a balanced CCR. The proband inherited from the father both the abnormal chromosome 5 resulting in segmental aneusomies of chr5 (loss) and chr18 (gain) and a der(2) homologue. Evidences suggest a chromoplexy mechanism for this CCR derivation, involving double-strand breaks (DSBs) repaired by non-homologous end joining (NHEJ) or alternative end joining (alt-EJ). The complexity of the CCR and the segregation of homologues elucidate the genetic model for this family. This study demonstrates the importance of combining multiple genomic technologies to uncover genetic causes of complex neurodevelopmental syndromes and to better understand genetic disease mechanisms.

## Introduction

Complex chromosomal rearrangements (CCRs) are intriguing genetic phenomena that challenge our understanding of the human genome’s structural integrity. These genomic DNA rearrangements involve intricate and often unexpected alterations in the organization of chromosomal material. CCRs represent a subclass of structural variations (SVs), distinct from simple translocations or unique locus simple DNA rearrangements, we operationally define these CCRs as involving at least three breakpoints on two or more chromosomes [1, 2].

In CCRs, multiple double-strand breaks (DSBs) are hypothesized to occur in different regions of the genome on two or more chromosomes, setting the stage for a complex genetic jigsaw puzzle [2, 3]. These genomic DNA breaks can be caused by various factors, including DNA damage or loss of genome integrity during DNA replicative repair mechanisms [3]. What ensues is a genomic reshuffling, characterized by the interplay of chromosome translocations, deletions, insertions, and inversions. This complicated choreography of genetic material challenges our conventional understanding of linear chromosomal sequences.

Understanding mechanisms for CCR derivation holds profound clinical significance, as they can be associated with a range of genetic disorders, including neurodevelopmental disorders (NDDs), and have implications for inheritance and for both family counseling and prognosis. Therefore, investigating CCRs is vital for both diagnosing and comprehending the genetic basis of these conditions. However, despite their clinical significance for proband and family, identifying CCRs remains a formidable challenge in the fields of genetics and clinical cytogenetics. The nature of these genome rearrangements, with multiple breakpoint junctions and various types of structural alterations, can challenge interpretations in the clinical diagnostic laboratory.

This study investigates a family with a female child proband who presented with a NDD, accompanied by subtle facial anomalies. Despite extensive prior investigations, her condition and the genetic model for the family remained an enigma, prompting a multifaceted technological exploration of her genomic landscape. The investigation initiated with a comprehensive examination of her genome, employing a multi-tier approach that included several genomic technologies, pointing to a CCR involving not one but three distinct chromosomal regions: 5p15.2, 2q31.3, and 18q21.32. We provide evidence implicating an underlying mechanism for the CCR, its clinical implications for the family, and the impact of gene dosage imbalance on the proband.

## Methods

### Patient enrollment

This study adhered to the principles of the Declaration of Helsinki. The family trio (proband, mother, and father) were enrolled under a protocol approved by the institutional review board (IRB) at Baylor College of Medicine (BCM) (H-29697). Written informed consent for publication of images was obtained. This family was identified through the Baylor–Hopkins Center for Mendelian Genomics (BHCMG) and BCM Genomics Research to Elucidate the Genetics of Rare Disease (BCM-GREGoR) database and as part of the analysis of a large Middle Eastern and North African (MENA) cohort with rare NDDs.

### Exome sequencing

Exome sequencing (ES) was performed at the Baylor College of Medicine Human Genome Sequencing Center (BCM-HGSC) with an Illumina dual indexed, paired-end pre-capture library per the manufacturer’s protocol with previously described modifications [4]. Paired-end sequencing was performed with the Illumina NovaSeq6000 platform. Samples achieved 98% of the targeted exome bases covered to a depth of 20× or greater and had a sequencing yield of 13.2 Gb. Illumina sequence analysis was performed with the HGSC HgV analysis pipeline, which moves data through various analysis tools from the initial sequence generation on the instrument to annotated variant calls (single nucleotide variants (SNVs) and intra-read insertions/deletions (indels)) [5, 6]. Copy number variation (CNV) analyses were implemented using read count from ES data that were normalized for log_2_ ratio calculation by the best reference approach using our in-house algorithm HMZDupFinder [7] and using XHMM (eXome-Hidden Markov Model) [8]. In the selection of control reference samples for log_2_ ratio calculation by HMZDupFinder, we implemented a strategy where the samples were chosen based on a minimum 10% correlation with probes designed for total genomic capture, as per protocols established in HMZDupFinder. This ensured that our controls were representative of the genomic variability assessed by the capture design, facilitating more accurate detection of duplications and deletions within the genome. For a detailed description of the log_2_ ratio calculation, refer to Du et al. [7].

### High-density array comparative genomic hybridization (aCGH)

High-resolution aCGH, using a 1 million probe whole-genome oligonucleotide microarray (Agilent microarray design ID:085903), was performed on all family members. All array-based experiments were implemented according to the Agilent aCGH protocol for probe labeling and hybridization with minor modifications [9].

### Nanopore long-read sequencing

Long-read genome sequencing (lr-GS) libraries were generated using the Oxford Nanopore technology (ONT) ligation sequencing kit and then sequenced with the PromethION Beta platform. Sequencing depth was calculated on the resulting alignments using mosdepth v0.2.38 with the parameters ‘-F 3588 -Q 1’, which calculated coverage of depth in 100 bp bins and included only primary and supplemental alignments. Alignment of long read DNA sequencing was performed with NGMLR v0.2.79 using default parameters along with the ‘-bam -fix’ parameter for long CIGAR string support. Average coverage of 30× was achieved for the trio.

### Breakpoint junction amplification and sequencing analysis

Soft-clipped reads overlapping breakpoint junctions were extracted from LR sequence alignment files and remapped to the human genome (GRCh38) with the UCSC BLAT tool to single base-pair resolution. Primers were designed upstream and downstream of the identified junction and polymerase chain reaction (PCR) amplification was performed using the HotStarTaq (Qiagen) polymerase with standard conditions. The amplified DNA rearrangement breakpoint junctions were confirmed by Sanger dideoxynucleotide sequencing.

## Results

### Case presentation

**Fig. 1:**
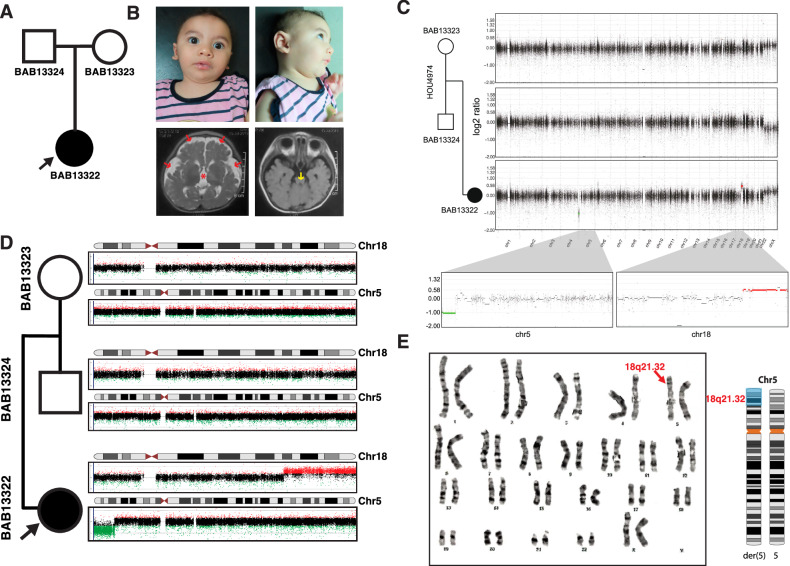
Chromosomes 5 and 18 copy number variants observed in child (BAB13322) with a neurodevelopmental disorder. **A** Pedigree structure with the father (BAB13324), mother (BAB13323), and proband (BAB13322). **B** Top row, photographs of female proband (BAB13322) highlighting mildly dysmorphic facial features. Bottom row, axial T2 (left) and T1-weighted (right) brain magnetic resonance imaging showing increased extra-axial cerebrospinal fluid (red arrows), ventricular dilatation (red asterisk), and thinning of the brainstem (yellow arrow). **C** Genome-wide log_2_ ratio of maternal (top plot) and paternal genome (middle plot) are consistent with a typical diploid genome or balanced translocation. Proband’s genome-wide log_2_ ratio (bottom plot) revealing abnormal log_2_ ratio signals indicating terminal deletion at 5p (green) and a terminal duplication at 18q (red). The segments corresponding to deletion and duplication are color coded: red indicates duplication (log_2_ ratio of 0.58) and green denotes deletion (log_2_ ratio of –1.0) which are presented in the zoomed in view providing a detailed view of the affected regions on 5p and 18q, respectively. **D** High-density array comparative genomic hybridization (aCGH) for the trio. Both parents showed diploid copy number (*N* = 2) for chromosomes 5 and 18, whereas the proband BAB13322 showed terminal 5p loss (green) consistent with deletion and terminal 18q gain (red) consistent with heterozygous duplication. These data suggested the possibility of an unbalanced translocation; t(5;18). **E** G-banded karyotype with chr5 karyogram image showing additional material (shaded blue) on derivative chromosome 5p (red arrow).

The female proband is the first child born to non-consanguineous healthy parents (29-year-old mother and 30-year-old father) at 39 weeks gestational age, by cesarean section (Fig. 1A, B). Her birth weight was 2.8 kg (–1.2 SD), length was 47 cm (–1.1 SD), and occipital frontal circumference was 31.5 cm (–2.2 SD). She was admitted to the neonatal intensive care unit for three days due to prolonged and marked jaundice.

At 9 months of age, this infant presented a clinical picture marked by a constellation of distinctive features. She exhibited global developmental delay, characterized by an inability to support her head and a lack of crawling abilities, as well as an inability to recognize her mother. On physical examination, anthropometric measurements showed a weight of 7.2 kg (–1.4 SD), a length of 66 cm (–1.3 SD), and a head circumference of 38.5 cm (–4.4 SD). Her facial features were distinctive but nonspecific for any recognizable pattern of human malformation: brachycephaly, a high forehead, arched eyebrows, epicanthic folds, wide palpebral fissures, upturned nose with a flat nasal tip, hypoplastic mandible and low-set large ears with elevated ear lobules. A unilateral single transverse palmar crease was noted, and neurological assessment showed axial hypotonia, mild limb hypertonia, and brisk reflexes. MRI showed a strikingly thin brain stem especially the pons, relatively small cerebellum in addition to increased extra-axial cerebrospinal fluid, mild supratentorial ventricular dilatation, and underdeveloped hippocampus (Fig. 1B). Additionally, the proband’s medical history is notable for congenital heart disease (CHD), specifically a patent ductus arteriosus (PDA) and patent foramen ovale (PFO), for which she was admitted at 2 months of age for a 20-day hospitalization. Cardiovascular evaluation by echocardiogram showed a PDA measuring 1.2 mm and a PFO also measuring 1.2 mm. Electroencephalogram (EEG) results remain within the normal range, reflecting typical brain electrical activity.

Initial G-banding analysis revealed a normal karyotype of 46,XX due to low resolution and an unclear banding pattern, highlighting the subjective nature of karyotyping and its reliance on the expertise of the examining cytogeneticist. Subsequent trio ES analysis did not identify evidence for disease-associated SNVs or indels.

### Copy number variation (CNV) analysis and validation

Subsequent analysis of the extant ES data, utilizing the HMZDupFinder [7] and XHMM tools [8] for the detection of CNVs, unveiled the presence of multiple seemingly de novo CNV deletions on chromosome 5p and duplications on chromosome 18q (Fig. 1C). To further interrogate these findings, a high-resolution aCGH was employed using a 1 million probe whole-genome oligonucleotide microarray (Agilent microarray design ID: 085903). Intriguingly, this advanced genome analysis demonstrated a diploid copy number status for chromosomes 5 and 18 in both parents, while the proband exhibited a terminal loss of 5p15.33–5p15.2 and a terminal gain on 18q21.32–18q23 (Fig. 1D). These molecular findings suggested the possibility of an unbalanced translocation t(5;18), a hypothesis corroborated by the subsequent repetition of karyotyping, which confirmed the presence of additional genetic material on 5p (Fig. 1E).

### Evidence for chromosome 2 interchromosomal insertion

**Fig. 2:**
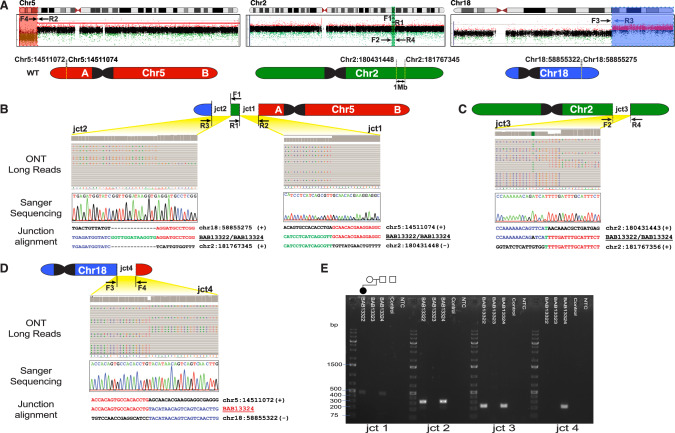
Breakpoint junctions (jct) with DNA recombinant joint visualized by multiple genomic technologies. **A** Long-read sequencing (Oxford Nanopore Technology, ONT), revealed apparent translocation (5;18) also included chromosome 2 genomic material (2q31.3) and provided evidence for a CCR between 5p15.2, 2q31.3, and 18q21.32. Ideograms (top) of chr5 (red), chr2 (green) and chr18 (blue). Beneath ideograms the aCGH data plots for chromosomes 5, 2, 18 showing the approximate location and direction- relative to the reference genome- of the PCR primers (forward, F; reverse, R) designed to capture the breakpoint junction sequences. In the bottom, simplified illustrations of chromosomes 5, 2, 18 showing the chromosomal coordinates (hg38) of the breakpoint junctions. **B**–**D** The four breakpoint junctions as visualized through each technology applied including long read whole-genome sequencing by ONT, Sanger dideoxy DNA sequencing, and breakpoint-junction alignment with reference human haploid genome. **E** Agarose gel electrophoresis for the PCR products of the four breakpoint junction sequences. Pedigree on top of the gel image is aligned with the band for each individual in the family. Amplification products of expected size (i.e., bands) are shown for junctions 1, 2, and 3 in the proband BAB13322 and father BAB13324 who had the CCR in a balanced state as evidenced by the fourth junction between 5p and 18q.

We performed trio long read ONT sequencing to unravel the architecture and the orientation of the translocated genomic regions. Surprisingly, breakpoint mapping revealed that the apparent translocation (5;18) also included chromosome 2 genomic material (1 Mb mapping to 2q31.3) and provided evidence for a CCR between 5p15.2, 2q31.3, and 18q21.32 (Fig. 2). Since the 2q31.3 material was translocated in a balanced state in the proband, ES read depth data showed no abnormalities in the proband’s chr2 ES data (Fig. S1). Moreover, none of the breakpoint sequences were detectable using the ES data given their location in deep intronic regions. ONT data also showed that the CCR was inherited from the father who has the CCR in a balanced state.

### Genomic rearrangement architecture and breakpoint junction sequences

ONT data revealed the presence of three distinct breakpoint junction sequences in both the proband and her father. Junction 1 connects the terminal portion of the deleted segment on chromosome 5p (Fig. 2A) with the proximal site of the inserted 2q31.3 genomic region; notably, 2q31.3 is connected in an inverted orientation (Fig. 2B). This junction presents a blunt end fusion between 5p and 2q31.3. The distal side of the 2q31.3 (chr2:181767345, hg38) is then connected to the beginning of the duplicated region in 18q in a direct orientation. Additionally, the junction reveals the insertion of a 14 bp segment (junction 2).

To investigate the third junction on chromosome 2, where the 2q31.3 segment was excised (Fig. 2C), we designed inward-facing primers. This junction exhibited a 1 bp microhomology. Since the father carries a balanced rearrangement, a fourth junction was exclusively detected in his ONT data. This junction connects the segment of 5p, which is deleted in the proband, with the 18q21.32 in an inverted orientation (Fig. 2D).

**Fig. 3:**
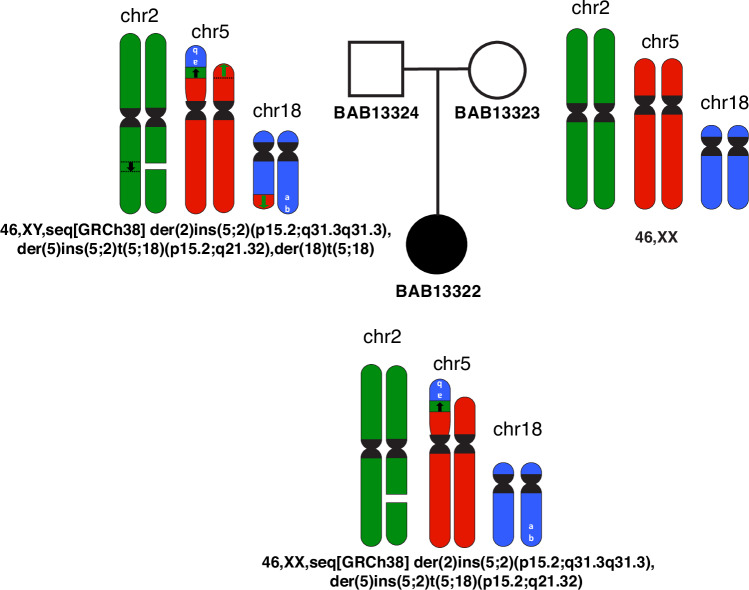
Complex chromosomal rearrangement segregation in family. Genomic material from different chromosomes is color coded: chr2 (green), chr5 (red), chr18 (blue); centromeres (black). Note deletion of one chr2 homologue (white space) and insertion into translocated chr18 on the chr5 short arm. Note the inverse orientation of the chr18 material translocated to the short arm of chr5 retaining the telomere structure (white a, b for orientation). Father BAB13324 showed a balanced CCR, mother BAB13323 showed a normal karyotype, whereas the proband BAB13322 had the inherited unbalanced CCR and segmental aneusomies of chr5 (loss) and chr18 (gain).

All breakpoints identified through ONT sequencing underwent validation via PCR amplification and subsequent Sanger sequencing. Gel electrophoresis of the PCR products (Fig. 2E) corroborated the inheritance of the CCR from the father, who maintains the translocation in a balanced state. This is evident from the presence of the fourth junction. Conversely, no bands were observed in the mother for any of the junctions, confirming the absence of the CCR in her genetic profile (Fig. 3).

### Chromoplexy complex genomic rearrangement

The CCR potentially occurred as a result of different DSBs that could be repaired by non-homologous end joining (NHEJ) or alternative end joining (alt-EJ) and be arranged into various derivative configurations: der(2), der(5), der(18). The full structure and the alignment of all the breakpoint junction sequences suggest chromoplexy as an underlying mechanism. Since the father carries the CCR in a balanced manner, possible chromosomal complement in the father’s cells is:

46,XY,seq[GRCh38] der(2)ins(5;2)(p15.2;q31.3q31.3),der(5)ins(5;2)t(5;18)(p15.2;q21.32),der(18)t(5;18).

NC_000002.12:g.180431443_181767356del

NC_000005.10:g.pter_14511074delins[NC_0000018.10:g.58855275_qterinv; GGTTGGATAAGGTG;NC_000002.12:g.180431448_181767345inv].

NC_0000018.10:g.58855322_qterdelins[NC_000005.10:g.pter_14511072inv].

At meiosis I, translocated chromosomes and their normal homologues synapse to form a quadrivalent. Therefore, unbalanced gametes are produced by adjacent segregation from a quadrivalent. Fertilization with a normal gamete produce the proband’s genome with unbalanced CCR:

46,XX,seq[GRCh38] der(2)ins(5;2)(p15.2;q31.3q31.3),der(5)ins(5;2)t(5;18)(p15.2;q21.32)

NC_000002.12:g.180431443_181767356del

NC_000005.10:g.pter_14511074delins[NC_0000018.10:g.58855275_qterinv;N[14];NC_000002.12:g.180431448_181767345inv].

## Discussion

Despite significant advancements in the fields of genetics and genomics and extensive research into the genetics of NDDs, many cases and families (~25–60%) remain unsolved [10–13]. There could be a multitude of reasons, from a genomic variation viewpoint, that underly the genetics contributing to these unresolved NDD cases. SVs represent a significant source of genetic variation in the human genome and collectively contribute to more inter-individual differences than SNVs [14]. On average, an individual’s genome is thought to contain 2.9 rare (<1%) coding SVs and ~2% of the population carries rare SVs larger than 1 Mb, including both balanced and complex rearrangements [15].

Genome sequencing (GS) studies, both short-read (sr-GS) and lr-GS, have revealed that complex genomic rearrangements (CGRs) are more abundant and diverse than previously recognized, comprising an estimated 2% of SVs in the human genome, with each human genome containing an average of 14 complex SVs [16]. Despite their significance, complex SVs are often overlooked during genomic analysis due to technical challenges associated with their identification and resolution of structure. Therefore, we hypothesize that CGRs may account for a significant proportion of unsolved NDD cases. The proband in this study exhibited a constellation of clinical features, including global developmental delay, craniofacial anomalies, and CHD. These clinical findings raised suspicions of an underlying genetic cause. While initial karyotyping and ES analysis did not reveal any single gene mutations, subsequent investigations enabled by lr-GS showed a CCR involving chromosomes 2, 5, and 18, further highlighting the importance of considering structural chromosomal abnormalities in cases of unexplained phenotypes. Initial G-banding analysis revealed a normal karyotype of 46,XX but potentially suboptimal resolution and an unclear banding pattern, highlighting the subjective nature of karyotyping and its reliance on the expertise of the examining cytogeneticist.

The der(5) contains a loss of 14.5 Mb in 5pter and a gain of 21.5 Mb from 18qter, in addition to a gain of 1.3 Mb from 2q. The calculated size differential, ~8.3 Mb, between the der(5) and normal chr5 may not be detectable by karyotyping; especially if the G-banding resolution is ≤550 bands. The detection of such small difference depends on banding quality and resolution as well as the changes in banding patterns. Retrospective examination of the 5p in the repeated karyotyping analysis assisted in the detection of this der(5).

Due to complexity of the genomic changes in this family, we chose lr-GS over sr-GS. This decision stems from the need for a thorough understanding of the connectivity of the CCR, particularly considering the limitations of sr-GS in accurately sequencing through human genome low-copy repeats and repetitive regions and the challenges of detecting large SVs.

**Fig. 4:**
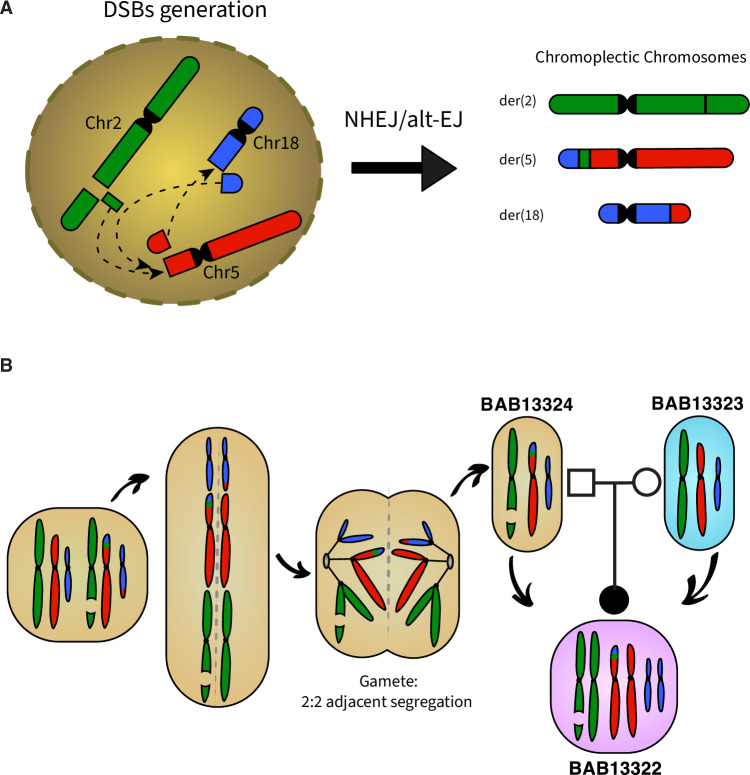
Complex chromosomal rearrangement: mechanism of formation. **A** Chromoplexy is a term that best describes the observed phenomenon. The CCR potentially occurring as a result of different DSBs that could be repaired by non-homologous end joining (NHEJ) or alternative end joining (alt-EJ) and be arranged into various derivative configurations: der(2), der(5), der(18). **B** The father contains balanced rearrangements including a translocation between 5p and 18q and an insertion of a 2q segment into the breakpoint between chromosomes 5 and 18. At meiosis I, translocated chromosomes and their normal homologues synapse to form a quadrivalent. Unbalanced gametes are produced by adjacent-1 segregation from a quadrivalent. Fertilization with a normal gamete derived from the mother produces the unbalanced complex chromosomal rearrangements in the proband that includes the derivative chromosome 5 with a 2q segment inserted between 5p and 18q and the derivative chromosome 2 with a deletion in 2q.

The mechanism(s) underlying CCR formation remains elusive; some studies propose models based upon the principle of parsimony and the minimum number of breaks required for the formation of the CCR [17, 18]. Since this CCR involves three chromosomes, the presence of blunt fusions, direct orientations, and inverted orientations in the junction sequences indicates that the rearrangement is highly complex, consistent with chromoplexy (Fig. 4A). It is a CGR mechanism that involves multiple DSBs occurring in different regions of the genome [19]. It often leads to a series of intricate chromosomal rearrangements and is characterized by the interplay of translocations, deletions, insertions, and inversions. The presence of multiple derivative chromosomes (der(2), der(5), and der(18)) within the described CCR, suggests that several DSBs occurred simultaneously or in close succession, resulting in the observed complex rearrangement or perhaps that a replicative repair through a nick might result in a one-ended DNA (oeDNA) molecule and a template switch between or within chromosome territories. The CCR’s involvement of chromosomes 2, 5, and 18 implies that these chromosomes were connected during the rearrangement process. Chromoplexy often forms multiple connections between different chromosomes, leading to the exchange of genetic material. This complexity arises from the diverse ways in which the DSBs are repaired, and the genomic regions are rearranged.

Our breakpoint data support the contention that NHEJ and/or alt-EJ mechanisms were involved in the repair of the DSBs. These mechanisms involve the direct joining of broken ends of DNA, often without the need for homologous sequences, and can lead to insertions, deletions, or inversions [20]. The father’s balanced rearrangement implies that NHEJ or alt-EJ was precise enough to create a stable, balanced configuration in his genome. However, the rearrangement became unbalanced when transmitted to the proband (Fig. 4B). This CCR complies with most aspects of chromoplexy, including the involvement of >2 chromosomes, the absence of sequence homology at their fusion breakpoints [19]. However, the fact that the breakpoints are not within transcriptionally active areas, which is usually the case in chromoplexy [19, 21], stands against our hypothesis of chromoplexy-type event. Chromoplexy, chromothripsis, and chromoanasynthesis are designated terms that describe the phenomenology of the observed genome changes. The term chromoanagenesis, i.e., chromosome rebirth, encompasses the phenomena of extensive rearrangement occurring in a single cell burst [19, 21, 22]. Chromothripsis could also be suggested as underlying mechanism of the CCR, given the lack of sequence homology at the breakpoints or only microhomology of a few nucleotides usually observed in chromothripsis similar to chromoplexy [21]. Chromothripsis and chromoplexy were first characterized in cancer genomes, however, they also have been shown to underlie Mendelian diseases and genomic disorders [23, 24].

Since the proband’s genome carries this CCR in an unbalanced manner, this suggests an altered gene dosage for specific genes and regions of 5p loss and 18q gain involved in the CCR; the observation of a normal 2q31.3 dosage and absence of one junction fragment present in the father is consistent with the segregation of the paternal homologue with the deleted 2q31.3. This imbalance in the proband leads to changes in the expression of the genes mapping within the 5p and 18q dosage-altered regions and can significantly impact the individual’s phenotype. The father, on the other hand, carries the same CCR but in a balanced state, which explains the absence of the clinical phenotype that is observed in the child. This contrast highlights the importance of maintaining the proper gene dosage for normal development and function [25].

Several dosage-sensitive genes have been identified in the 5p region that may contribute to the proband’s phenotype. For example, five genes (*TERT*, *SEMA5A*, *MARCH6*, *CTNND2*, *NPR3*) are classified as dosage sensitive leading to haploinsufficiency [26]. Perhaps *TERT* haploinsufficiency could contribute to perturbations of telomere biology underlying some CCR. It is firmly established that the 5p deletion is responsible for Cri du Chat syndrome (MIM#123450), characterized by a distinctive cat-like cry [27], while the duplication of 18q leads to Edwards syndrome [28, 29]. These are rare chromosomal syndromes, each associated with distinct physical and mental impairments. However, our case presents a unique clinical scenario, showcasing a blended phenotype that may exhibit some overlap with both Cri du Chat and Edwards syndrome. This includes distinctive facies, involving a high forehead, arched eyebrows, epicanthic folds, wide palpebral fissures, and a hypoplastic mandible. These features are consistent with those seen in Cri du Chat syndrome and share some similarities with the facial features seen in Edwards syndrome [30], but the blending or mixture precludes diagnosis of either; particularly in the context of an initial “normal karyotype”. Global developmental delay, axial hypotonia, and other neurological findings are also observed in both Cri du Chat and Edwards syndrome. Moreover, the child has a history of CHD, specifically a PDA and PFO. While CHD is not a hallmark feature of Cri du Chat syndrome, it can be present in some cases [30].

Examination of the chromosomal coordinates for all breakpoint junctions, along with their flanking regions (±500 kb), using 3D Genome Browser (http://3dgenome.fsm.northwestern.edu/index.html) human B-cells Hi-C data, were used to determine if these breakpoints fell within topologically associating domain (TAD) or TAD boundaries. Also, we investigated whether any regulatory elements could have been affected by the CCR. Our analyses showed that all breakpoints were situated within TADs, surrounded by regulatory elements (Fig. S2). The finding that all breakpoints are situated within TADs indicates that the CCR likely occurs within the context of these higher-order chromatin structures. This suggests that the rearrangement may not necessarily disrupt entire TADs but rather occur within the boundaries of existing TADs. However, it’s important to note that the rearrangement could still influence gene regulation within these TADs through mechanisms such as TAD fusion or “position effect”, where regulatory elements are brought into close proximity with different genes due to changes in chromatin conformation. Furthermore, the presence of regulatory elements surrounding the breakpoints implies that the CCR may have the potential to affect gene regulation within these TADs. This could result in altered expression patterns of genes located nearby, leading to potential phenotypic consequences. Overall, these findings provide valuable insights into the spatial organization of the genome and the potential functional implications of the CCR on gene regulation and chromatin structure. The analysis highlights the importance of considering TADs and regulatory elements when investigating chromosomal rearrangements and their effects on genome function.

Taken together, the case stands as an exemplar of the impact of ES analyses for CNVs using the freely available bioinformatic tools such as XHMM, HMZDelFinder, and HMZDupFinder [7, 8, 31]. Incorporation of such analyses has demonstrated a significant increase in diagnostic rates within clinical settings [4, 32, 33], as has scoring ES data for Runs of Homozygosity (ROH) using the Absence of Heterozygosity as a surrogate measure of ROH [10, 34]. Moreover, this case shows both the intertwined nature of chromosomal rearrangements and complexity of genome mutation, underscoring the importance of employing a diverse array of genomic technologies to untangle the consequences for both patient and family.

## Supplementary information

Figure.S1

Figure. S2

Figure S1 and Figure S2 Legends

## Data Availability

Microarray data generated in this study are available through GEO under the accession number GSE265815. BAM files for the proband indicating the specified structural variants are deposited in the Sequence Read Archive (SRA), accession number PRJNA1104271.
