# Supplementary figures and images for: Genomic Balancing Act: deciphering DNA rearrangements in the complex chromosomal aberration involving 5p15.2, 2q31.1, and 18q21.32
Source: Eur J Hum Genet. 2024 Sep 10;33(2):231–8. doi: 10.1038/s41431-024-01680-1 (PMC11840051; doi:10.1038/s41431-024-01680-1)

BAB13323

HOU4974

BAB13324

BAB13322

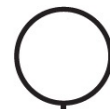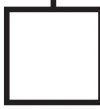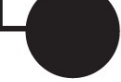

log2 ratio

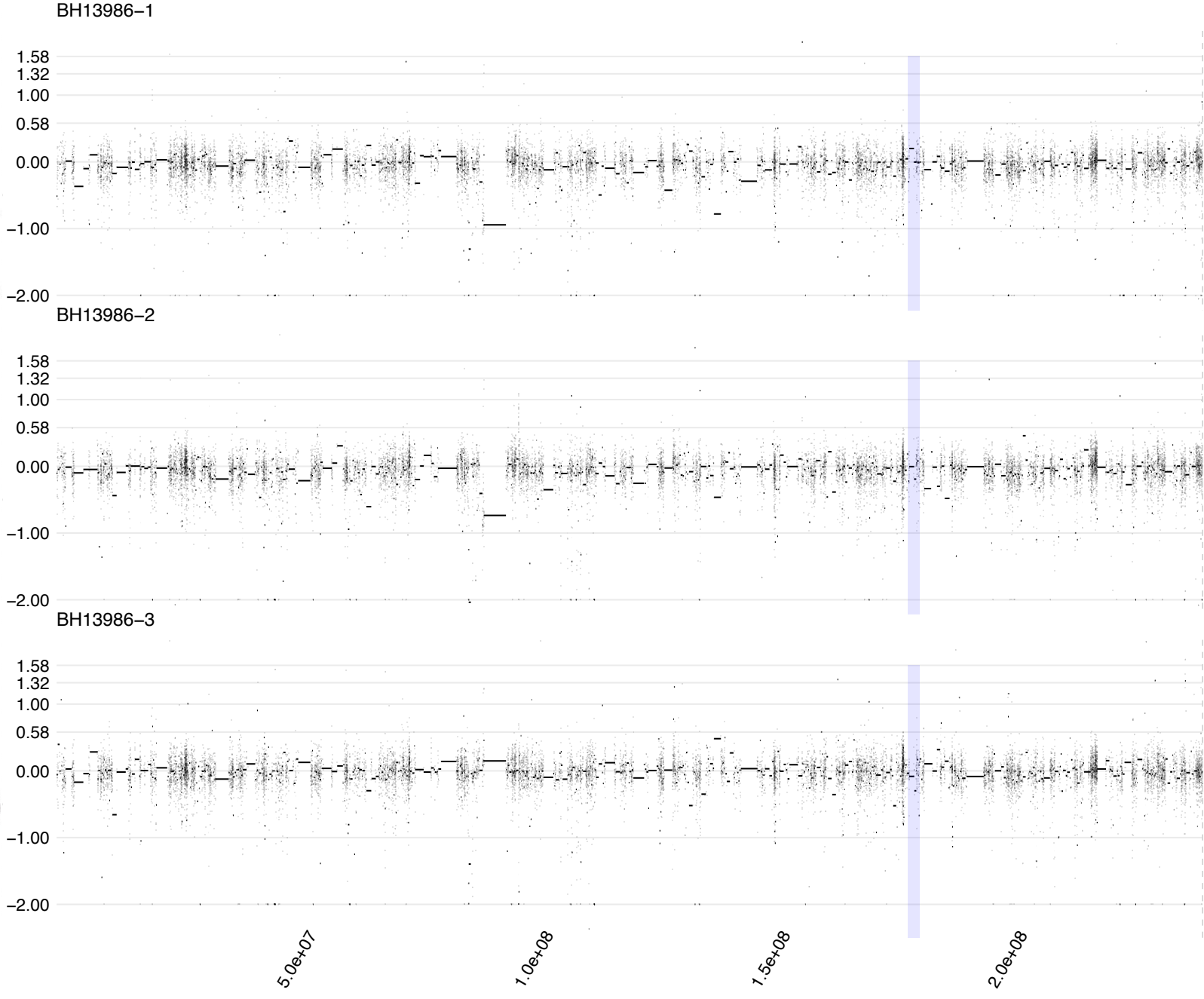

Supplement: Supplementary file 1 — Figure.S1 [file 41431_2024_1680_MOESM1_ESM.pdf]

Chr5:14511072-Chr5:14511074

WT

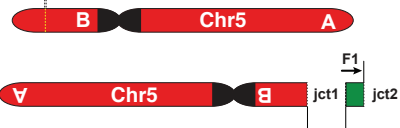

Chr2:180431448 Chr2:181767345

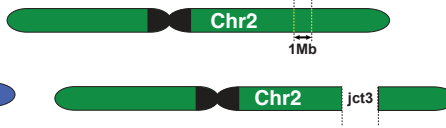

Chr18:58855322-Chr18:58855275

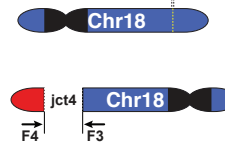

A

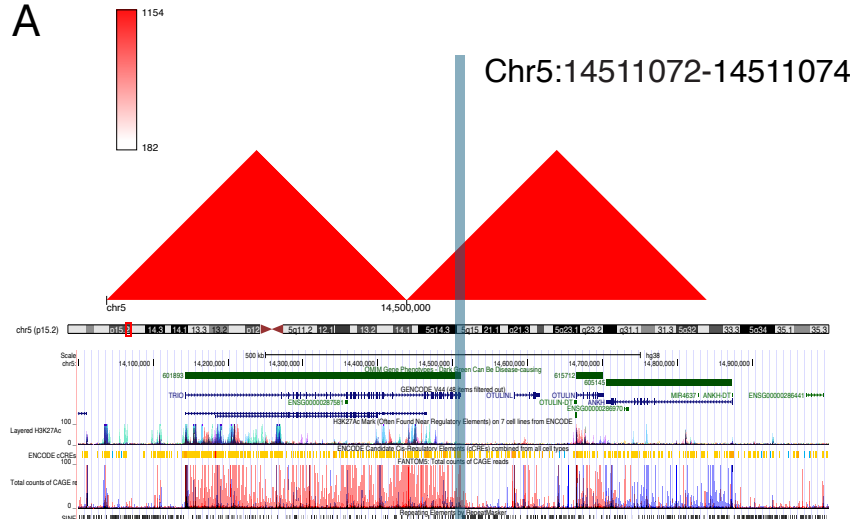

B

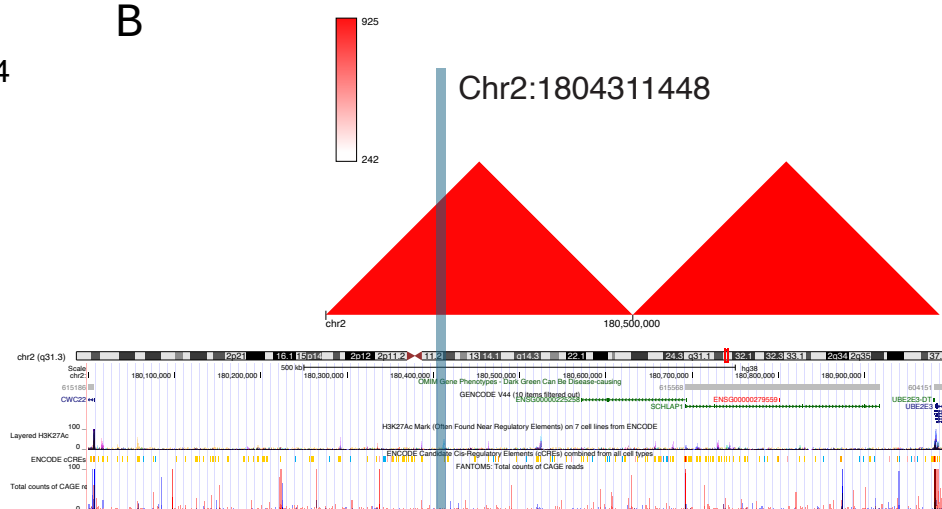

C

Chr2:181767345

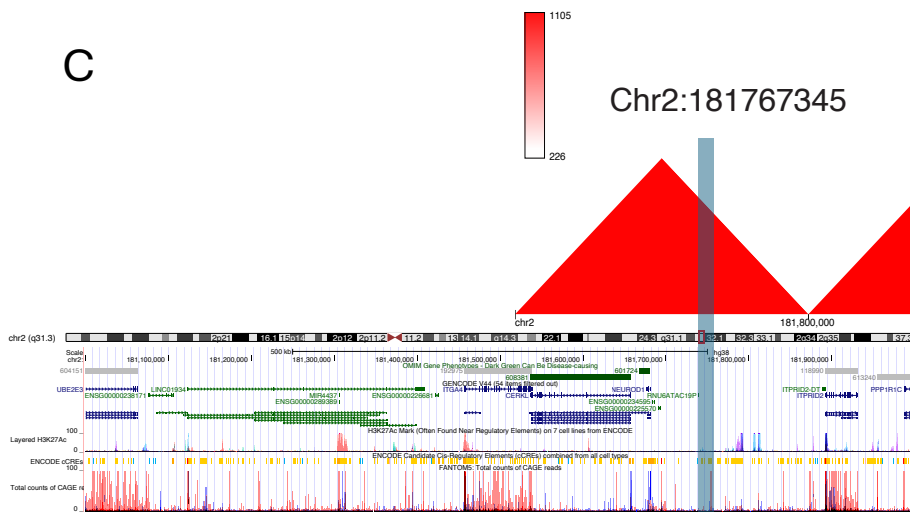

D

Chr18:58855275-58855322

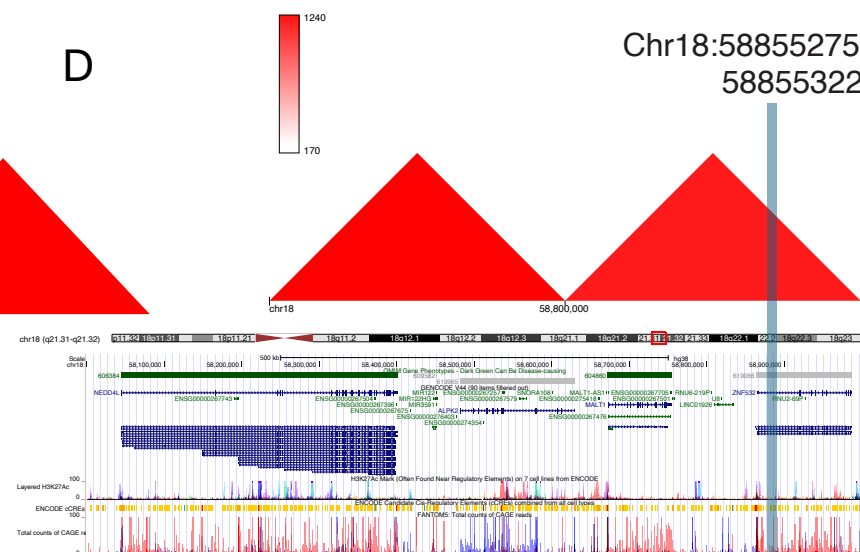

Supplement: Supplementary file 2 — Figure. S2 [file 41431_2024_1680_MOESM2_ESM.pdf]
