# Supplementary material for: Genomic Balancing Act: deciphering DNA rearrangements in the complex chromosomal aberration involving 5p15.2, 2q31.1, and 18q21.32
Source: Eur J Hum Genet. 2024 Sep 10;33(2):231–8. doi: 10.1038/s41431-024-01680-1 (PMC11840051; doi:10.1038/s41431-024-01680-1)
Supplement: Supplementary file 3 — Figure S1 and Figure S2 Legends [file 41431_2024_1680_MOESM3_ESM.docx]

**Figure S1. Chr2 read depth data shown with short read ES data by HMZDupFinder.** Log2 ratio of paternal and proband genome consistent with diploid genome of chr2.

**Figure S2. Breakpoint junctions Topological associated Domains (TADs) and regulatory elements in the human ES cell Genome.** All chromosomal breakpoints (A-D) reside within TADs and flanked by enriched regulatory regions and known OMIM disease genes.
